# Case Report: Pulsed field ablation for epicardial right-sided accessory pathway

**DOI:** 10.3389/fcvm.2024.1392264

**Published:** 2024-04-26

**Authors:** Jana Haskova, Petr Peichl, Eva Borisincova, Josef Kautzner

**Affiliations:** Department of Cardiology, Institute for Clinical and Experimental Medicine, Prague, Czech Republic

**Keywords:** accessory pathway, radiofrequency ablation, pulsed field ablation, intracardiac echocardiography, electroanatomical mapping

## Abstract

We present a case of a 32-year-old male with a history of palpitations and preexcitation on ECG who underwent altogether four failed catheter ablations using different approaches in the two other electrophysiology centers within two years. ECG showed overt preexcitation with a positive delta wave in lead I and negative in leads V1–V3, suggesting a right free wall accessory pathway. During the electrophysiological study, the accessory pathway was localized on the free lateral wall. However, the electrograms and mapping during atrial and ventricular pacing suggested the presence of true epicardial accessory pathway. Repeated radiofrequency energy delivery with the support of the steerable sheath and excellent contact (as assessed by intracardiac echocardiography) at the earliest ventricular activation was not successful. Therefore, the Farawave catheter (Boston Scientific, Inc) was used, and a flower configuration with the intention to cover the entire atrial attachment of the pathway during ventricular pacing was selected. Application of pulsed field resulted in interruption of accessory pathway conduction. An electrophysiological study one year later confirmed the persistent effect of ablation. This case illustrates the potential utility of pulsed field energy for the ablation of atrial insertion of the accessory pathway with an epicardial course. Such an approach can avoid epicardial mapping and access and may improve the safety of the procedure.

## Introduction

Accessory pathway ablation is highly successful in experienced centers, making it the therapy of choice for patients with Wolff-Parkinson-White syndrome (1). Some rare failures are attributed to anatomically unusual pathways or their epicardial locations (2–6). Epicardial accessory pathways are often located in the posterior septal space, connecting via muscular bands around the coronary sinus or its tributaries. Rarely, the pathway connects the right atrial appendage with the right ventricle or is located lower on the tricuspid annulus lateral side (7–11). Catheter ablation of these pathways may be challenging.

## Case description

A 32-year-old male with a history of palpitations and preexcitation on ECG underwent altogether four failed catheter ablations in the two other electrophysiology centers between 2020 and 2022. Different strategies were used, including access to the right atrium via jugular vein. The patient was implanted with an implantable loop recorder (Biomonitor, Biotronik), which showed multiple episodes of orthodromic AV re-entrant tachycardia. Over time, arrhythmias became more frequent, and the patient was referred for a re-ablation to our center.

**Figure 1:**
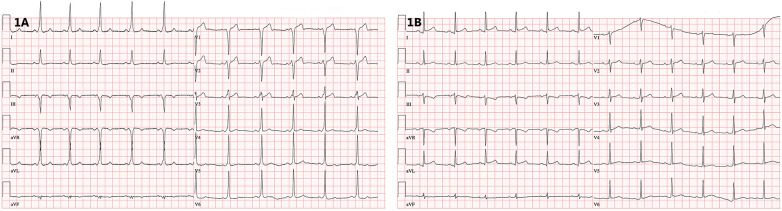
(**A**) 12-lead ECG showing ventricular preexcitation with a negative delta wave in the right precordial leads. (**B**) 12-lead ECG after successful catheter ablation documenting loss of preexcitation (recorded at 25 mm/s).

His physical examination was normal. ECG showed overt preexcitation with a positive delta wave in lead I and negative in leads V1–V3, suggesting a right free wall accessory pathway (Figure 1A). Echocardiography revealed a non-dilated left ventricle with a normal ejection fraction and borderline thickness of the interventricular septum (11 mm). Besides a trivial tricuspid regurgitation, no valvular heart disease was observed, and no signs of pulmonary hypertension were present.

## Diagnostic assessment and therapeutic intervention

An electrophysiology study was performed under conscious sedation. Both femoral veins were used to introduce diagnostic catheters into the coronary sinus, the His bundle region, and the right ventricle or atrium. Finally, an intracardiac echocardiography probe (Acuson AcuNav 10F, Siemens Medical Solutions) was introduced to the right atrium to monitor the ablation catheter location and tissue contact. The electroanatomical mapping system (CARTO 3, Biosense Webster, Inc) was employed to support the procedure. Point-by-point mapping with 4 mm tip was used (Thermocool catheter, Biosense Webster).

**Figure 2:**
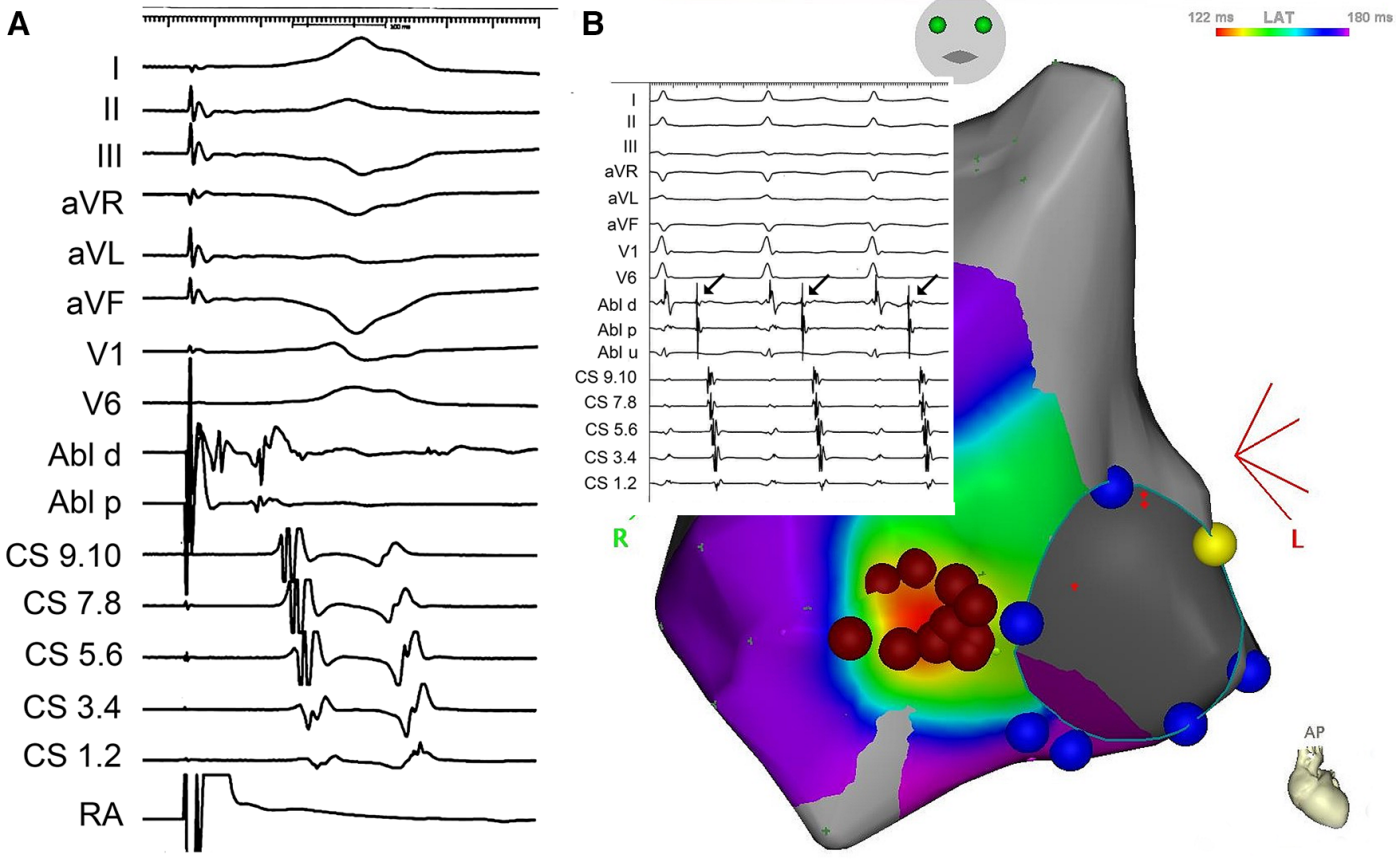
(**A**) Local signals from the ablation catheter (ABLd and ABLp) at the site of the earliest ventricular activation during pacing in the right atrium (RA). Upper part shows surface ECG leads, lower part signals from the coronary sinus). (**B**) Electroanatomical activation map of the right atrium during right ventricular pacing, documenting the earliest activation remotely from the tricuspid annulus (red dots mark radiofrequency ablation cloud with the central tag removed to depict early activation). Inset depicts local electrogram at the tricuspid annulus during orthodromic AV reentry.

**Figure 3:**
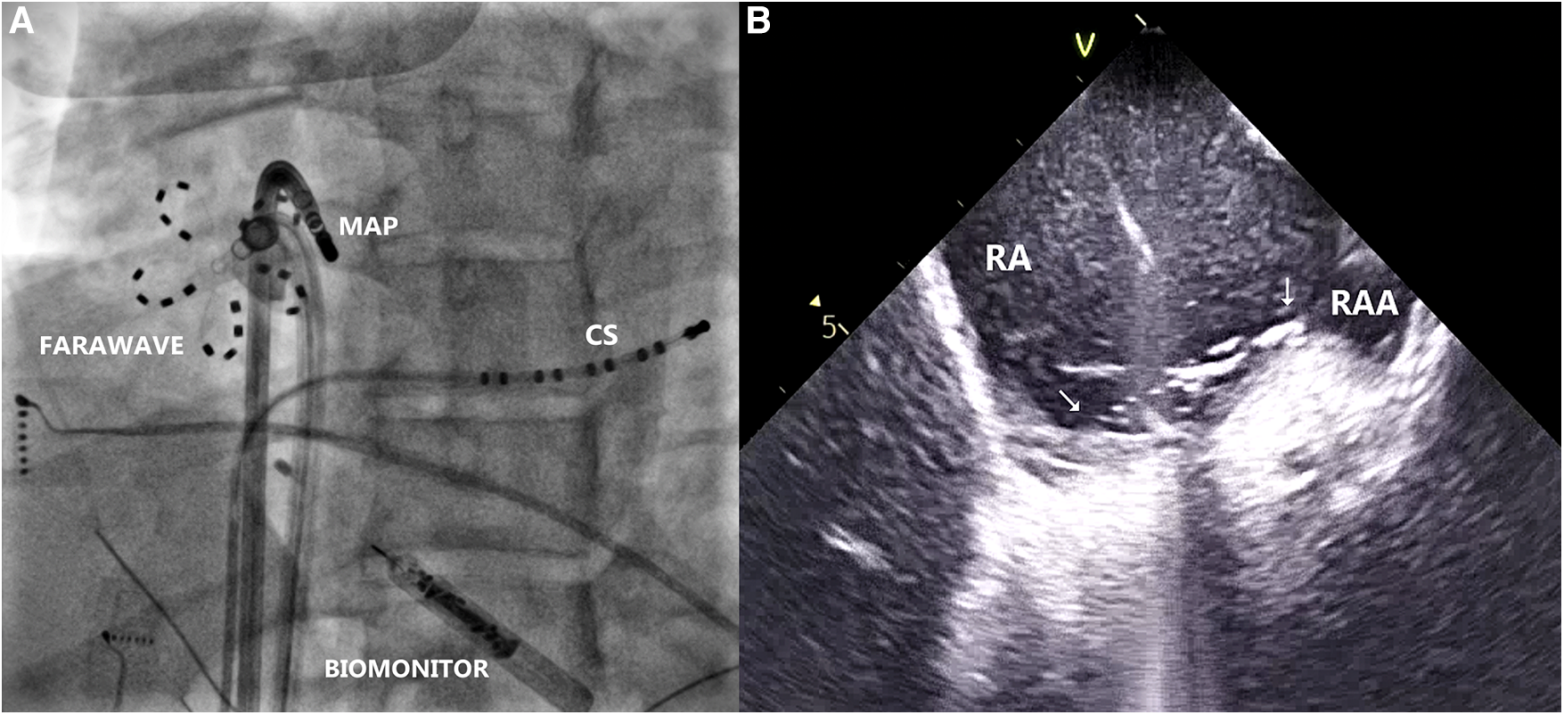
(**A**) Radiogram in left anterior oblique projection, showing farawave catheter at the site of the earliest atrial activation during ventricular pacing, parallel to mapping catheter (MAP). Decapolar catheter is introduced in the coronary sinus (CS). Biomonitor is visible in lower part of the image. (**B**) Intracardiac echocardiogram depicting location of the Farawave catheter and its contact with the tissue at the ablation site (arrows). RA, right atrium; RAA, right atrial appendage.

During sinus rhythm, the PQ interval was 108 ms, AH interval 76 ms, and HV interval 22 ms; the antegrade and retrograde refractory periods of the accessory pathway were 375 ms and 344 ms, respectively. An orthodromic AV reentry with cycle length of (CL) 400 ms could be readily induced. The procedure was complicated by episodes of atrial fibrillation that were easily inducible by catheter manipulation and that required electrical cardioversion. Rapid mapping around the tricuspid annulus confirmed the lateral location of the pathway. However, in the position of the earliest ventricular activation (−29 ms) during atrial pacing, the AV interval was not very short, and no electrograms of interest could be recorded (Figure 2A). Mapping below the tricuspid valve revealed a relatively large area of early activation. Ablation at the site the earliest ventricular activation (35 W/30 ml/min, 60 s) had no effect. During ventricular pacing, the earliest atrial activation was localized remotely from the tricuspid annulus (15 mm), suggesting true epicardial accessory pathway (Figure 2B). Repeated radiofrequency energy delivery with the support of the steerable sheath and excellent contact (as assessed by intracardiac echocardiography) at this spot was not successful (30–35 W/15 ml/min, up to 90 s). Therefore, the decision to change ablation strategy was made. The Farawave catheter (Boston Scientific, Inc) was used, and a flower configuration with the intention to cover the entire atrial attachment of the pathway was selected (Figures 3A,B). Application of pulsed field resulted in immediate interruption of accessory pathway conduction (Figure 1B). However, the conduction recurred repeatedly within minutes. After 14 deliveries, the effect persisted for thirty minutes. Subsequent electrophysiology study showed 1:1 conduction through the AV node up to 150 beats per minute (400 ms). Adenosine administration resulted in a transient AV block without accessory pathway conduction. No ST segment elevations or other complications were noted.

The next day, the preexcitation pattern transiently reappeared on ECG. However, during subsequent follow-up, the patient had normal ECG without preexcitation and no arrhythmic events. The effect persists for more than one year. In December 2023, the patient underwent repeated EP study for short episodes of palpitations and documented short runs of supraventricular tachycardia on the implantable monitor. No preexcitation was documented as well as no retrograde conduction. Adenosine administration resulted in a transient complete AV block. No arrhythmia was inducible by programmed atrial or ventricular stimulation, including isoprenaline administration.

## Discussion

Our case illustrates that pulsed field ablation could be used even in rare cases of epicardially located accessory pathways. To the best of our knowledge, this is the first documented case of epicardial accessory pathway ablation using pulsed field energy. After ineffective radiofrequency ablation at the area of atrial insertion of the pathway, the Farawave catheter was used in the flower configuration, placed across this region, and repeated pulsed field energy delivery interrupted accessory pathway conduction.

Right-sided accessory pathways with epicardial course are considered resistant to conventional ablation (6–9). Some groups described the value of electroanatomic mapping to accurately localize the atrial insertion sites of these accessory pathways and facilitate catheter ablation (9, 10). Chen et al, reported on a series of eleven patients mapped with electroanatomic mapping system and successfully ablated after previously failed one or more procedures. Atrial insertion was separated from the tricuspid annulus by an average of 14.3 ± 3.9 mm, and the local activation time was 27.8 ± 17.0 ms earlier than that of the corresponding annular point. Radiofrequency ablation at the site of the earliest atrial activation was successful. Another strategy of electroanatomic mapping was described by Fishberger et al. (11), in which a microcatheter was placed in the right coronary artery as a roadmap to facilitate the quick and accurate location of the accessory pathway.

Regarding the strategy of catheter ablation, one option could be cryoablation, which also solves the problem of catheter instability because of catheter adherence (12). Bipolar radiofrequency ablation may be effective and safe in cases of posteroseptal accessory pathways potentially of epicardial location, which are resistant to conventional unipolar radiofrequency ablation from endo- and epicardium (13). Other groups suggested percutaneous epicardial mapping and ablation (14, 15). Alternative approaches, such as a superior venous access/approach and/or reverse loop within the right ventricular inflow, were already used in previous ablation attempts. We discussed with the patient the possibility of epicardial mapping and ablation against the attempt with pulsed field energy. The patient preferred the latter solution. Regarding the selection of a pulsed field delivery tool, we believed that the use of a large footprint catheter to ablate the entire area of atrial insertion of the pathway would be preferable to the use of a solid tip catheter. These were the only available options available to us at that time. Having a fair experience with the Farapulse system both for ablation of atrial fibrillation and typical atrial flutter (several hundred cases at the time of this procedure) with an excellent safety profile, we considered this approach as reasonable option. Especially, taking into account four previous failed ablation procedures. The choice likely worked, blocking area of atrial tissue that serves as an entrance to the accessory pathway rather than hitting epicardial fibers itself. In our previous experience with a few similar cases, we achieved exclusion of the atrial insertion area by circumferential radiofrequency ablation around the earliest atrial activation during ventricular pacing. Interestingly, it appears that pulsed field energy may need some time to achieve lesion maturation since the conduction through the pathway transiently reappeared the next day after ablation.

In conclusion, this case illustrates the potential utility of pulsed field energy for the ablation of atrial insertion of the accessory pathway with an epicardial course. This approach can avoid epicardial mapping and access and may improve the safety of the procedure.

## Patient perspective

From a patient's point of view, the described solution was accepted against the alternative of epicardial mapping and ablation. More than one year after the procedure, the patient has no complaints, and his ECG remains without preexcitation.

## Data Availability

The raw data supporting the conclusions of this article will be made available by the authors, without undue reservation.
