# Supplementary material for: Case Report: Pulsed field ablation for epicardial right-sided accessory pathway
Source: Front Cardiovasc Med. 2024 Apr 26;11:1392264. doi: 10.3389/fcvm.2024.1392264 (PMC11082373; doi:10.3389/fcvm.2024.1392264)
Supplement: Supplementary file 2 [file Table1.docx]

**Timeline**

Primary manifestation documented AVRT and pattern of preexcitation

5 months first ablation attempt

2 years and 7 months second ablation attempt

2 years and 8 months third ablation attempt

2 years and 10 months fourth ablation attempt

2 years and 11 months index ablation procedure

4 years control electrophysiology study, no conduction via pathway
